# Correction: Bio-conjugation of graphene quantum dots for targeting imaging

**DOI:** 10.1039/c9ra90067e

**Published:** 2019-09-30

**Authors:** Fei Jia, Shuyu Lv, Sha Xu

**Affiliations:** School of Chemical Engineering, Changchun University of Technology Changchun 130012 China jiafei19861112@163.com

## Abstract

Correction for ‘Bio-conjugation of graphene quantum dots for targeting imaging’ by Fei Jia *et al.*, *RSC Adv.*, 2017, **7**, 53532–53536.

The authors regret that the versions of Fig. 2 and 4 displayed in the original article were incorrect. The loading dye in Fig. 2c should be the artificial marker for molecular weight analysis. The original Fig. 4 did not support the EGFR binding due to the cell endocytosis, and the result has been revised with an improved data set. The correct versions of Fig. 2 and 4 are shown below.

**Fig. 2:**
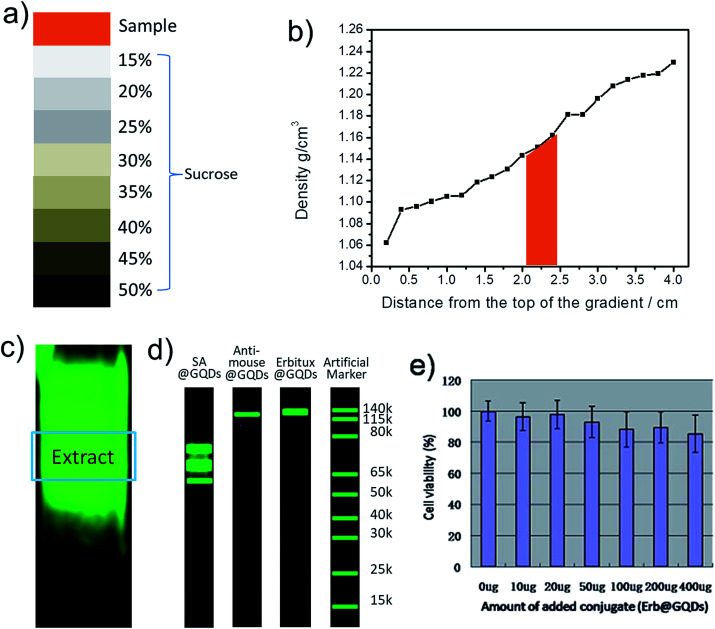
(a) The sucrose DGU gradient used for purifying the conjugates. (b) The desired SA@GQDs positions in the DGU column. (c) PL image of the DGU column after ultracentrifuge. (d) Bolt 4–12% Bis–Tris gel electrophoresis analysis of SA@GQDs, anti-mouse@GQDs and Erbitux@GQDs, respectively. The loading dye with confirmed molecule weight was tested as references. (e) The cell viability of conjugate Erbitux@GQDs (SCC cell).

**Fig. 4:**
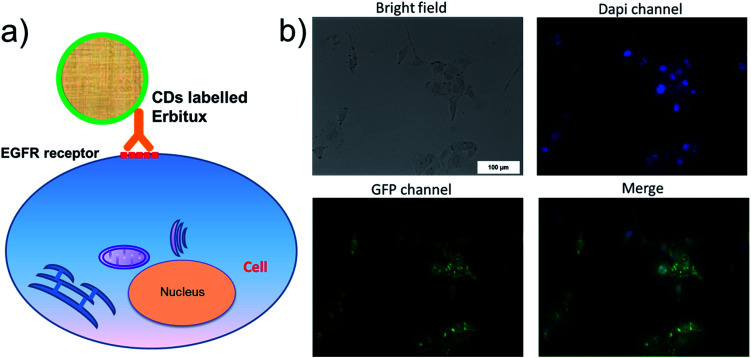
(a) SCC cell imaging by Erbitux@GQDs which is binding to the EGFR receptor of SCC cell membrane. (b) The bright field, and PL picture at different channels. After merging the PL picture of Dapi and GFP channels, the successful staining of membrane and nucleus was observed. The quantum yield of GQDs is much lower than Dapi, and we have to tune the contrast/brightness to reach the same quality between Dapi and GQDs. The emission of Dapi also has some signal in GQDs channel, and we have re-draw the data to get rid of the Dapi signal.

The Royal Society of Chemistry apologises for these errors and any consequent inconvenience to authors and readers.

## Supplementary Material

